# Higher Ramie mosaic virus transmission efficiency by females than by males of *Bemisia tabaci* MED

**DOI:** 10.1038/s41598-019-57343-5

**Published:** 2020-01-16

**Authors:** Jing Peng, Gang Xie, Songbai Zhang, Limin Zheng, Yang Gao, Zhuo Zhang, Luyun Luo, Pin Su, Dongwei Wang, Yong Liu, Liangying Dai, Deyong Zhang

**Affiliations:** 1 Plant Protection college, Hunan Agricultural University, No 1 Nongda Road, Furong District, Changsha, 410120 Hunan province P.R. China; 2 Hunan Plant Protection Institute, No 726 Second Yuanda Road, Furong District, Changsha, 410125 Hunan Province P.R. China

**Keywords:** Behavioural ecology, Evolutionary ecology

## Abstract

Begomoviruses can modify their transmission vector, *Bemisia tabaci*, to benefit their spread, although this may not always be the case. Here, the new begomovirus *Ramie mosaic virus* (RaMoV) and its vector *B. tabaci* MED, which is dominant in China and many regions of the world, were used as a model to examine direct and indirect interaction and virus transmission by *B. tabaci* MED of different sexes. No significant direct or indirect effects of RaMoV were observed in *B. tabaci* MED females, although RaMoV could shorten the life span of *B. tabaci* MED females by up to 4 days. A test of RaMoV transmission by different sexes of *B. tabaci* MED showed that there was higher virus transmission efficiency by females than males. Overall, RaMoV is transmitted by *B. tabaci* MED in a sex-dependent manner, and further research is needed to uncover the mechanism of the difference in RaMoV transmission by different sexes of *B. tabaci*.

## Introduction

Approximately 80% of epidemic plant viruses in the field are transmitted by insect vectors from plant to plant and spread to distantly located regions in this way^1^. The whitefly *Bemisia tabaci* (Gennadius) (Hemiptera: Aleyrodidae) is a species complex with a worldwide distribution, and at least 35 cryptic species are widely recognized based on the evidences of molecular phylogenetic data and reciprocity between genetic groups^2^. Of these cryptic species, two invasive species, Mediterranean (MED) and Middle East-Asia Minor 1 (MEAM1), severely harm plants by direct feeding and by indirect transmitting plant viruses^3^.

Plant viruses are not passively transmitted by their vectors; instead, they can produce direct and indirect effects on the vectors to modify their behaviour, life span and fitness to benefit their own transmission. *Tomato yellow leaf curl virus* (TYLCV) could directly mediate the behaviours of its vector, whitefly, including settling, probing and feeding, to enhance its transmission efficiency^4,5^. In the case of a plant virus indirectly affecting its vector, the fecundity, longevity and population density of whiteflies increased when feeding on *Tomato yellow leaf curl virus* (TYLCV)-infected tobacco plants^6–9^.

In addition to the direct and indirect mutual relationships of plant viruses and their vectors, plant virus transmission efficiency by whitefly is relatively different depending on sex. Compared with males of *B. tabaci* MEAM1, females possessed higher TYLCV transmission efficiency on tomato plants^10^. A similar sex-based difference in transmission efficiency was also found in *B. tabaci* MED^11^.

The capacity of vectors to acquire, retain, and transmit plant viruses is critical information for inferring plant virus epidemiology^12^. Although the characteristics of acquisition, retention and transmission of several plant viruses in the genus *Begomovirus* by *B. tabaci* have been well documented^11,13–15^, the detailed characteristics of the begomoviruses transmitted by *B. tabaci* still require additional study to understand the outbreaks of *Begomovirus* in the world.

Ramie mosaic virus (RaMoV) is a new bipartite begomovirus that was documented from infected *Boehmeria leiophylla*^16,17^. Plant viruses in the genus *Begomovirus* are important pathogens in the tropical and sub-tropical regions of the world, are exclusively transmitted by whitefly in a persistent-circulative mode^18^ and severely damage a wide range of economic crops, such as tomato, pepper and tobacco^19^. It is rational to deduce that RaMoV would be a potential threat to important crops.

In recent years, *B. tabaci* MED has progressively become a dominant species in China^20–23^; thus, in this study, direct and indirect effects were examined using the RaMoV and *B. tabaci* MED model. In addition, the acquisition, retention and transmission of RaMoV by the different sexes of *B. tabaci* MED was compared. The results would scientifically evaluate the potential epidemiology of RaMoV in the field.

## Result

### Symptoms of RaMoV-infected tobacco plants

**Figure 1:**
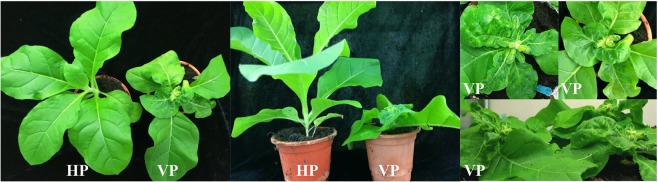
Symptoms of RaMoV-infected (VP) tobacco plants (10 dpi).

To determine the indirect effects of RaMoV on *B. tabaci* MED, RaMoV-infected tobacco plants were produced. Tobacco plants with 6 true leaves were inoculated with RaMoV in the 3rd leaf by *Agrobacterium tumefaciens* containing an infectious clone of RaMoV. At 10 dpi, compared with healthy tobacco plants, RaMoV-infected tobacco plants were severely stunted and lacked apical dominance. The new leaves of RaMoV-infected tobacco plants manifested shrivelling and mosaic symptoms (Fig. 1).

### Direct and indirect effects of RaMoV on longevity and fecundity of *B. tabaci* MED

**Figure 2:**
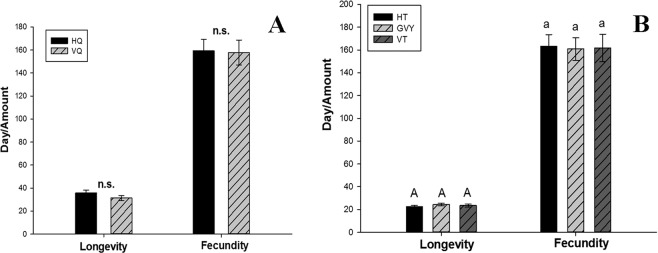
Direct and indirect effects of RaMoV on longevity and fecundity of *B. tabaci* MED. (**A**) Direct effects on *B. tabaci* MED by RaMoV; HQ: non-viruliferous *B. tabaci* MED; VQ: viruliferous *B. tabaci* MED; (**B**) Indirect effects on *B. tabaci* MED by RaMoV; HT: healthy tobacco; GVY: tobacco inoculated with *Agrobacterium tumefaciens* without RaMoV plasmid; VT: RaMoV-infected tobacco.

To determine the direct effects of RaMoV on females of *B. tabaci* MED, viruliferous and non-viruliferous females of *B. tabaci* MED fed on healthy cucumber plants, which are not natural hosts of RaMoV. As shown in Fig. 2, although the life span of the viruliferous females of *B. tabaci* MED was 4 d shorter than that of the non-viruliferous females of *B. tabaci* MED (31–35 d), the difference was not significant (*F*_1,80_ = 0.503, *P* = 0.480). Figure 2 also revealed that no significant difference (*F*_1,80_ = 0.533, *P* = 0.467) in the fecundity of females of *B. tabaci* MED was associated with RaMoV (Fig. 2A).

The indirect impacts on fecundity and longevity of females of *B. tabaci* by RaMoV were examined via three treatments with non-viruliferous females of *B. tabaci* MED on healthy tobacco plants, RaMoV-infected tobacco plants and tobacco plants inoculated with *A. tumefaciens* without the RaMoV plasmid. As Fig. 2 shows, the mean number of eggs (*F*_2,96_ = 0.012, *P* = 0.988) and the longevity (*F*_2,96_ = 0.672, *P* = 0.513) of *B. tabaci* MED females were not significantly different among the three treatments (Fig. 2B).

### Acquisition of RaMoV DNA by different sexes of *B. tabaci* MED

**Figure 3:**
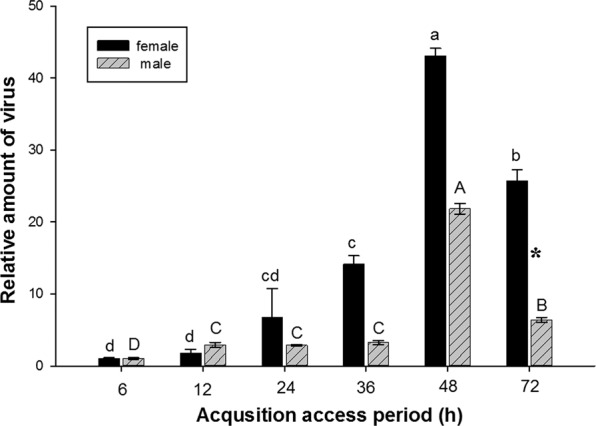
Acquisition of RaMoV DNA by *B. tabaci* MED gender.

The RaMoV acquisition capability of different sexes of *B. tabaci* MED was compared. RaMoV DNA attained maximal viral loads in both females and males of *B. tabaci* MED at 48 h AAP (*F*_female 5,12_ = 78.817, *P* = 0.002) (*F*_male 5,12_ = 419.587, *P* < 0.001) and decreased after 48 h. During the whole AAP in which *B. tabaci* acquired RaMoV, the females acquired more virus than the males; the difference was not significant in the 6–48 h AAPs (*P* > 0.05) and was significant at 72 h AAP (*P* < 0.05) (*F*_1,4_ = 9.259, *P* = 0.038) (Fig. 3).

### Retention of RaMoV DNA by different sexes of *B. tabaci* MED

**Figure 4:**
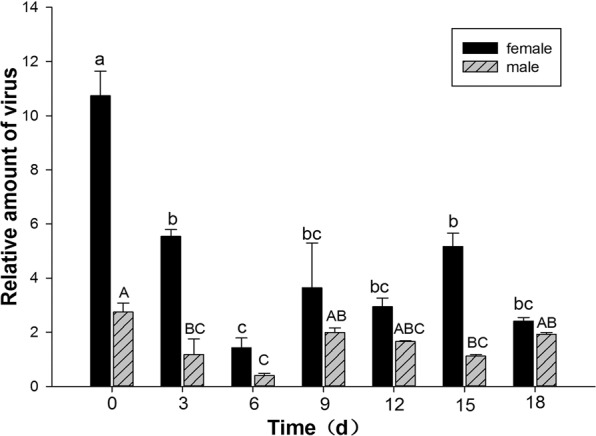
Retention of RaMoV DNA by different sexes of *B. tabaci* MED.

As *B. tabaci* MED loads maximal RaMoV DNA at 48 h AAP on RaMoV-infected tobacco, the female and male populations of *B. tabaci* MED fed on RaMoV-infected tobacco for 48 h were then transferred to healthy cucumber plants. At day 0, the relative amount of RaMoV DNA retained in females was greater than in males of *B. tabaci*. The RaMoV DNA amount plunged from day 0 to day 6 (*F*_female 6,14_ = 16.380, *P* < 0.001) (*F*_male 6,14_ = 8.030, *P* = 0.001) and equilibrated from day 9 to day 18 in both sexes of *B. tabaci*. The retained RaMoV DNA in females was always higher than that in males of *B. tabaci*, and the difference was significant from day 12 to day 15 (*F*_12day 1,4_ = 11.868, *P* = 0.026) (*F*_15day 1,4_ = 11.700, *P* = 0.027) (Fig. 4).

### Transmission of RaMoV DNA by different sexes of *B. tabaci* MED

**Figure 5:**
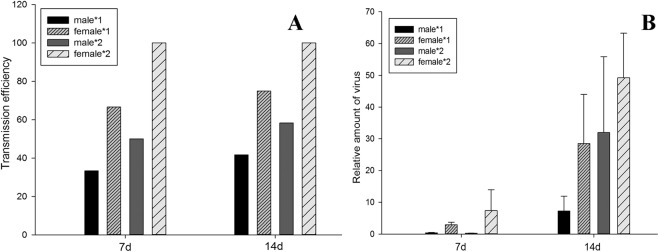
Transmission of RaMoV by DNA by different sexes of *B. tabaci* MED. (**A**) Transmission efficiency of RaMoV by different sexes of *B. tabaci*; (**B**) Relative amount of RaMoV DNA in tobacco leaves transmitted by different sexes of *B. tabaci*.

The transmission efficiency of RaMoV by different sexes of *B. tabaci* MED was determined, and RaMoV-infected tobacco was verified by conventional PCR. As shown in Fig. 5A, the transmission efficiency of RaMoV by females was higher than by males of *B. tabaci* MED. The transmission efficiency of RaMoV by one female could reach 66.67% when fed on tobacco for 7 d and 75% for 14 d, while that by two females could reach 100% for both 7 d and 14 d. However, lower than 40% transmission efficiency of RaMoV to tobacco by one male of *B. tabaci* was observed for 7 d or 14 d.

The relative amount of RaMoV DNA accumulation in tobacco leaves inoculated by *B. tabaci* of different sexes was estimated. As shown in Fig. 5B, virus DNA accumulation in tobacco leaves infested by females of *B. tabaci* MED was much higher than by males. The highest difference was up to 33.25-fold virus DNA accumulation in tobacco leaves exposed to two females compared to two males of *B. tabaci* MED at 7 d. However, the virus DNA accumulation in the tobacco leaves was not significantly different among the four treatments (one male, two males, one female, two females) after 7 d (*F*_3,26_ = 0.984, *P* = 0.416) and 14 d (*F*_3,26_ = 1.400, *P* = 0.262).

## Discussion

It is well known that plant viruses have co-evolved with their transmission vectors and benefited via enhanced virus transmission^4,24^. Nevertheless, distinct effects of certain plant viruses on their vectors exist, and such interactions are ambiguous^25^.

This study revealed, using a new bipartite begomovirus, RaMoV, and its vector, *B. tabaci* MED, as a model, that there is no significant direct or indirect effect of RaMoV on females of *B. tabaci* MED (Fig. 2). This result is reminiscent of another bipartite begomovirus, *Tomato mottle virus* (ToMoV), which also had no significant effect on the fertility and longevity of its whitefly vector^26^. However, the begomovirus *Tomato yellow leaf curl china virus* (TYLCCNV) could significantly decrease the fertility and longevity of females of *B. tabaci*^6^. The direct and indirect effects of RaMoV and its vector, females of *B. tabaci* MED, together with a previous study, reconfirmed the disparate interactions of plant viruses and their vectors.

Our data show that both females and males of *B. tabaci* are able to acquire, retain and transmit RaMoV, and the ability of females to acquire and retain the RaMoV DNA was significantly higher than that of males (Figs. 3, 4). This indicates that females could transmit more RaMoV DNA to host plants than males of *B. tabaci* MED, and the transmission efficiency results (Fig. 5) also verified this tendency. This result was consistent with those achieved for other begomoviruses and their vectors: that *B. tabaci* transmits begomovirus in a sex-dependent manner, and higher transmission efficiency achieved by females^11,27,28^. Females acquired and retained much more RaMoV DNA than males of *B. tabaci* MED, which was possibly dependent on the feeding capacity of females being higher than that of males of *B. tabaci*^4^; however, the relationship between *B. tabaci* feeding behaviour and its virus acquisition ability still needs to be confirmed.

In conclusion, this study showed that a new begomovirus, RaMoV, has no significant direct or indirect effect on its vector, *B. tabaci* MED, that RaMoV transmitted by *B. tabaci* MED in a sex-dependent manner, and that higher transmission efficiency is shown by females. Further research is needed to uncover the mechanism of the difference in transmission of RaMoV by *B. tabaci* of different sexes.

## Material and Methods

### Insects, plants and RaMoV clone

Tobacco (*Nicotiana tabacum* cv. Samsun NN) and cucumber (*Cucumis sativus* L. cv. ChangChunMiChi) were grown in a potting mix in 1.5 L pots (one plant/pot) at 26 ± 2 °C under a 16 h light/8 h dark day cycle. The tobacco plants at the 6–8 true-leaf stage was inoculated with virus by *A. tumefaciens* GV3101 containing a RaMoV plasmid, which was constructed and stored in our laboratory.

The *B. tabaci* MED population was maintained in whitefly-proof screen cages at 26 ± 2 °C with a 16 h light/8 h dark day cycle. The purity of the MED population was monitored by CAPS (cleavage amplified polymorphic sequence) of *mtCOI* (mitochondrial cytochrome oxidase I genes) with the restriction endonuclease *VspI*^29^.

### Fecundity and longevity of whiteflies

The direct effects of RaMoV on the fecundity and longevity of females of *B. tabaci* were examined by clip-caging viruliferous or non-viruliferous insects with leaves of healthy cucumber. Viruliferous insects were generated from new-born female adults fed on the leaves of RaMoV-infected tobacco for 48 h by clip-caging and then transferred to leaves of healthy cucumber. Non-viruliferous insects were generated on healthy tobacco of the same growth stage. A set of 40 female *B. tabaci* individuals in each experimental group was used.

The indirect impacts of RaMoV on the fecundity and longevity of female *B. tabaci* MED were examined by clip-caging newly emerged non-viruliferous *B. tabaci* from healthy cucumber plants with healthy tobacco, RaMoV-infected tobacco or *Agrobacterium*-infected tobacco. The clip-cages were attached to the third to sixth leaf of each tobacco plant. Each newly emerged non-viruliferous *B. tabaci* MED adult female was clip-caged to ovipositor on tobacco for five days (one clip-cage per tobacco plant) until death.

The eggs deposited on cucumber/tobacco leaves were counted and marked with an insect needle under a stereomicroscope (Leica, DFC450, Leica Microsystes, Germany). The adults and the clip-cages were then moved to new leaves. Every female was checked every 5 days until death, and their longevity was calculated to determine the effects of RaMoV for each replicate.

### Acquisition, retention and transmission of RaMoV by different sexes of *B. tabaci* MED

To test the acquisition of RaMoV by different sexes of *B. tabaci* MED, approximately 300 newly emerged (0–24 h) non-viruliferous female or male adult individuals were taken from their respective cultures and transferred to feed on RaMoV-infected tobacco plants enclosed in a clip-cage. To ensure that the insects remained healthy, the transfer process of insects from leaf to leaf was performed gently. Following adult transfer, we randomly collected 20 adults from the leaves of the two plants at the end of 6 designated acquisition access periods (AAPs; 6 h, 12 h, 24 h, 36 h, 48 h, 72 h). The collected adults were stored at −20 °C and later assayed individually for detectable RaMoV DNA by qPCR.

To evaluate the retention of RaMoV by *B. tabaci* MED on the basis of sex, approximately 300 newly emerged (0–24 h) non-viruliferous female or male adults were transferred to feed on two RaMoV-infected tobacco plants enclosed in an insect-proof cage for 48 h AAP to obtain 100% viruliferous *B. tabaci* MED. Then, the viruliferous adult female or male *B. tabaci* MED were collected and released to feed on a healthy cucumber plant, a non-host of RaMoV. After the initial release, a group of 20 live adults were collected at 0, 3, 6, 9, 12 and 15 d. The insect samples collected were stored at −20 °C, and later, RaMoV DNA was detected by qPCR.

To estimate the transmission of RaMoV to tobacco plants by *B. tabaci* MED on the basis of sex, viruliferous adults generated as above were collected and inoculated in groups of 1 and 2 on the second leaf from the bottom of an uninfected tobacco plant at the three-true-leaf stage using a clip-cage for a 48 h inoculation access period. After 7 d and 14 d, the tobacco plants in each of the treatments were examined for virus infection by RaMoV symptoms and conventional PCR, and the RaMoV DNA accumulation in tobacco leaves was quantified by qPCR.

### Viral DNA detection in *B. tabaci* and tobacco

Total genomic DNA of *B. tabaci* and tobacco was extracted by the CTAB method^30^. For specific detection of RaMoV in *B. tabaci* and tobacco, the specific primer (CP-F: 5′-TATCGCAAGCCCAAGATG-3′ and CP-R, 5′-3′: GACCTCCAGTAACAGTTGACG) of the partial CP gene fragment of RaMoV (774 bp) (GenBank No. NC_010791.1) was used to conduct conventional PCR. The reaction mixture (20 µL) contained 10 × PCR buffer (no Mg^2+^) 2 µL, MgCl_2_ (50 mM) 0.8 µL, dNTPs (10 mM) 0.2 µL, primers (10 mM) 0.2 uL each, enzyme mix (1000 U) 0.5 µL, RNA inhibitor 0.1 µL, ddH_2_O 16 µL. The following cycling conditions were used: initial denaturation at 94 °C for 2 min; 35 cycles of 94 °C for 30 s, 55 °C for 1.5 min, and 72 °C for 1.5 min; and a final extension step at 72 °C for 10 min. The PCR products were sequenced to ensure the accuracy of the insertion sequence.

For quantification of RaMoV DNA in insects and tobacco, qPCR was conducted as in a previous study^13^ with minor modifications. The specific primers (qPCR-F: GGTTCTGCGTAAAGTCCG and qPCR-R: TTGACCTCCAGTAACAGTTGAC) for the partial CP gene fragment of RaMoV (200 bp) (GenBank No. NC_010791.1) were used. qPCR reactions were carried out in a 96-well optical plate in an Analytik Jena AG PCR instrument, and the accompanying software was used for qPCR data normalization and quantification. Amplifications for RaMoV were performed with 2 × TransStar Green qPCR Super Mix UDG (Trans Gene Biotech (Beijing), Co., Ltd). For each sample, three replicates were performed for each of the three biologically independent experiments. The relative gene expression of the RaMoV CP gene was calculated using the 2^−△△Ct^ method^31^. β-actin from *B. tabaci*^13^ and EF-1a^32^ from tobacco were used as reference genes to normalize the gene expression level.

### Data analysis

Statistical analyses were performed with SPSS (version 22.0; SPSS Inc., Chicago, IL, USA). Means were compared by the least significant difference (LSD) test at P = 0.05.
